# HOME vs. LAB hair samples for the determination of long-term steroid concentrations: a comparison between hair samples collected by laypersons and trained research staff

**DOI:** 10.1007/s00702-021-02367-3

**Published:** 2021-07-20

**Authors:** Nadine Skoluda, Isabell Piroth, Wei Gao, Urs M. Nater

**Affiliations:** 1 Department of Clinical and Health Psychology, Faculty of Psychology, University of Vienna, Vienna, Austria; 2 Delegated Psychologist, TherapieRaum, Hunzenschwil, Switzerland; 3 Chair of Biopsychology, Faculty of Psychology, Technical University of Dresden, Dresden, Germany

**Keywords:** Hair steroid, Hair cortisol, Hair cortisone, Hair progesterone, Hair DHEA, Hair collection

## Abstract

Hair segment analysis is a valuable tool for the assessment of cumulative long-term steroid secretion. Preliminary findings suggest comparable cortisol concentrations in hair collected by instructed laypersons and research staff. However, it remains unclear whether hair sample quality and hair steroids other than cortisol are affected by level of experience (laypersons vs. research staff), home collection circumstances (instructions, familiarity to participant, performance confidence), and characteristics of the layperson (conscientiousness). Sixty participants (23.6 ± 3.9 years; 43 females) provided hair samples twice: first collected by laypersons (HOME) according to provided instructions (written vs. written/video-based instructions) and second by trained research staff (LAB) on the same day or the day after the HOME collection. Hair steroid concentrations (cortisol, cortisone, DHEA, progesterone) were determined using LC–MS/MS. Hair sample quality was evaluated using nine predefined criteria. Laypersons completed questionnaires for the assessment of potential factors of hair outcome measures (hair steroid concentrations, hair sample quality). Hair steroids from HOME and LAB samples were positively correlated (*rs* between 0.76 and 0.89) and did not significantly differ, with the exception of cortisone. The quality of hair samples was significantly higher for LAB than for HOME samples. Neither HOME collection circumstances nor layperson-related characteristics had an impact on hair outcome measures. However, a low self-reported performance confidence predicted a high absolute difference between HOME and LAB DHEA. In summary, our findings suggest higher quality of hair samples collected by trained research staff compared to instructed laypersons. However, these differences might be negligible, considering the high correlation between HOME and LAB hair steroid concentrations, with the characteristics of the layperson or collection circumstances having a minor impact on hair steroids and hair sample quality. These findings provide further support for the notion that well-instructed laypersons can be enabled to collect hair samples.

**Supplementary Information:**

The online version contains supplementary material available at 10.1007/s00702-021-02367-3.

## Introduction

Hair analysis has a long history in the field of forensics, toxicology, and drug and doping control due to the retrospective assessment of long-term incorporation and storage of various substances in the hair shaft (e.g., Cooper et al. 2012). It provides the ideal tool to assess accumulated steroid secretion, such as cortisol, over prolonged periods of time, which cannot be covered (or only with great effort) by classical measures using fluid media such as blood and saliva (snapshot measures) or urine (usually 12- or 24-h urine collection). Hair has advantages over these biological matrices due to its easy and non-invasive assessment, long-term stability, and low storage requirements (for more details see reviews, Russell et al. 2012; Stalder and Kirschbaum 2012).

Hair collection is commonly performed by trained research or medical staff (e.g., research assistants, study nurses) according to standardized procedures, for which participants usually agree to come to the laboratory. However, certain populations of interest are not easily accessible due to their limited flexibility or mobility (e.g., home caregivers, rural population), restrictive circumstances (e.g., lockdown or self-quarantine due to pandemics), or in the context of wide area sampling in epidemiological studies, due to high logistical challenges on either side (lab appointments, home visits). A possible solution to this problem is to enable laypersons to collect hair samples in the participant’s home.

Ouellet-Morin et al. (2016) were the first to address this issue by creating an adapted hair collection kit including collection materials (curved scissors, hair clamps, collection card), a hair characteristics questionnaire, and written and illustrated instructions, which can be mailed back and forth between the laboratory and the participant’s home. For their validation study, thirty-four adolescents were asked to collect a hair sample at home, with the help of a familiar person and the hair collection kit. The same participants were invited to the laboratory the following week, where a trained research assistant collected a second hair sample. Hair cortisol concentration (HC) was determined. Preliminary findings were promising, with high correlations between (*r* = 0.91, *p* < 0.001) and no group differences in (*t* = 0.06, *p* = 0.95) HC collected at home and at the laboratory. Enge et al. (2020) chose an experimental approach to replicate previous findings in an adult sample (Sample 2: *N* = 50, age range: 19–58 years). In brief, within one lab session, participants provided hair samples twice: In a randomized order, a partner (using written and video-based instructions) and a trained experimenter collected hair samples from each participant. Again, there were high correlations and no significant differences in HC between samples collected by the partner and the trained experimenter (*r* = 0.84, *p* < 0.001; *F*(1,49) = 1.46, *p* = 0.206). In a subsample (*n* = 36), Big Five personality traits of the partner who collected the hair sample did not act as a moderator between HC and collection method (partner vs. trained experimenter).

Taken together, previous findings support the notion that HC is not affected by the experience or personality traits of the individual who collects hair samples. However, it remains unclear whether these findings are generalizable to hair steroids other than HC. Assessments of the quality of the collected hair sample have been neglected so far, even though high quality of the hair sample constitutes a crucial prerequisite for the quality of the hair analysis and may thus affect the measurement of hair steroid concentrations. Furthermore, it remains unclear whether and how the format of instructions, circumstances of collection, and personality of the layperson who collects the hair sample may affect hair outcomes.

Therefore, the current study builds on and addresses the unresolved open questions of the two previously published studies (Enge et al. 2020; Ouellet-Morin et al. 2016). First, we increased the sample size (*N* = 60). Second, we determined various hair steroids (i.e., cortisone, DHEA, progesterone) in addition to HC. Third, considering that hair is continuously growing (Loussouarn et al. 2005), the collection of the HOME and LAB samples^1^ was scheduled on the same day or one day apart, with the first sample always being collected by a layperson at the participant’s home. In contrast to previous studies, we compared the quality of hair samples collected by laypersons (HOME) and trained research staff (LAB), because good quality of the hair sample substantially enhances the quality of hair analysis and, ultimately, of the analytes under question. To investigate the effect of instructions on hair sampling, we compared written instructions with and without an instruction video. Lastly, we studied the impact of circumstances for HOME collection and laypersons’ characteristics on hair outcome measures. For example, it is conceivable that individuals are particularly good at collecting hair samples if they are well acquainted and familiar with the other person, feel confident about their own performance, and are generally conscientious.

Based on the previous findings and research gaps, we hypothesize a high correlation (1.1) and no group differences (1.2) in hair steroid concentrations between HOME and LAB samples, collected by laypersons and research staff, respectively. Further, we hypothesize that the hair quality will be rated higher for LAB compared to HOME hair samples due to the experience of the trained research staff (1.3). We explore whether hair steroid concentrations differ between HOME and LAB samples, depending on the format of instructions (2.1) and the quality of the hair sample (2.2). Additionally, we explore how laypersons evaluate instruction materials (2.3) and their own performance confidence (2.4), depending on the instructions they have received. Finally, we examine whether the magnitude of the difference in hair steroid concentrations between HOME and LAB samples (3.1), and the quality of the HOME hair sample (3.2), is associated with layperson-related characteristics and circumstances of HOME collection. We examine whether layperson-related characteristics and circumstances of HOME collection may affect the strength of the association between HOME and LAB hair steroid concentrations (3.3).

## Methods

### Sample

Using G*Power (Faul et al. 2007), we calculated that a total sample size of at least 46 participants was needed, assuming a large effect size of the correlation (correlation *p* H1 = 0.5 against correlation H0 = 0), alpha error = 0.01, and power of 0.90. A total of 60 participants were recruited to account for possible dropouts and exclusion due to extreme values. The participants (mean age ± *SD*: 23.6 ± 3.9 years; 43 females; mean BMI ± SD: 21.8 ± 2.2 kg/m^2^) were recruited using advertisement boards at the University of Marburg and public places in Marburg, Germany, between November 2016 and January 2017. Participants were required to fulfill the following inclusion criteria: age between 18 and 35 years, neither underweight nor obese (17 ≤ BMI ≤ 30), no regular smoking (≤ 7 cigarettes/week), neither pregnancy nor breastfeeding, no self-reported chronic physical or mental disorders, hair length of at least 3 cm, fluent in written and spoken German, and knowing a person who is willing to collect a hair sample from the participant.

The sixty *laypersons* (mean age ± SD: 25.8 ± 8.7 years, range 18–64 years; 36 females) were required to have no previous experience of hair collection, to not work in a hair-related profession (e.g., hairdresser), to have unimpaired eyesight, and to be fluent in written and spoken German to understand and follow the instructions. Twenty-one of the laypersons were in a romantic relationship with the participant and a further three were related to the participant (grandmother, mother, twin).

Three trained *research staff members* (mean age ± SD: 33.0 ± 10.0 years, range: 23–43 years; all female) collected hair samples at the laboratory. They had at least 1 year of experience in collecting hair samples from adults.

## Procedures

After successfully completing the telephone-based screening (see inclusion criteria, 2.1), participants were randomly assigned to one of the two instruction conditions: (A) written instructions only, *n* = 30, or (B) written and video-based instructions, *n* = 30 (see Hair collection kit and instructions). Participants and laypersons provided written informed consent and were compensated with 16 and 8 Euros, respectively. Participants received a hair collection kit and instructions via mail or picked them up from the Department of Psychology (University of Marburg, Germany). Laypersons conducted the first hair collection at the participant’s home (HOME) and documented the hair collection by completing a prepared form and taking a photograph of the back of the participant’s head indicating the collection site of hair samples. Laypersons were asked to provide information about their relationship to the participant, and to evaluate the provided instructions and their own performance. The second hair sample was collected and documented by a trained research staff member at the laboratory (LAB) on the same (*n* = 49) or the following day (*n* = 11). Furthermore, participants, laypersons, and research staff were asked to complete validated and self-developed questionnaires (see Questionnaires, Suppl. S1).

### Hair collection kit and instructions

The hair collection kit contained: three hair clamps of different sizes, a comb, barber’s scissors, several loop threads (100% polyester) for fixating hair strands, aluminum foil in which to wrap hair samples, and a permanent marker for labeling (date of sampling, participant’s code) and for marking the scalp-near end on the foil. The written instructions contained an illustrated step-by-step guide. Laypersons in the B condition were additionally provided with a CD and an online link to an instruction video (available online at: https://www.youtube.com/watch?v=8Jf_aIDtz4o&feature=youtu.be), which was the same video as that used by Enge et al. (2020).

### Questionnaires

*Participants* were asked to complete an online survey on stress, health, and protective factors (Unipark survey, QuestBack GmbH, Cologne, Germany) at home prior to hair sampling, and to complete questionnaires about sociodemographic and hair characteristics on the day of hair collection (see Suppl. S1 for a complete list of questionnaires and descriptive statistics).

*Laypersons* completed paper-and-pencil questionnaires on the day of the HOME assessment (see Suppl. S1). Laypersons completed the German version of the NEO Five-Factor Inventory (NEO-FFI) (Borkenau 1991; Costa and McCrae 1992); however, only the conscientiousness subscale was of interest in the current analysis. On a 10-cm visual analogue scale, laypersons rated how familiar they are with the participant, assuming that high familiarity between participant and layperson increases trust and confidence in the layperson during the collection procedure. Furthermore, they were asked to evaluate the instructions in terms of helpfulness and comprehensibility on a five-point Likert scale, and their own performance confidence on a 10-cm visual analogue scale (‘All in all, how confident do you feel that you have collected the hair sample properly according to the instructions?’).

### Hair steroid concentrations

Hair steroid concentrations [cortisol (HC), cortisone (HCn), DHEA (HDHEA), progesterone (HProg)] were determined in the first 3 cm proximal hair segment to the scalp, which retrospectively reflects the last 3 months (Wennig 2000). The hair washing, steroid extraction procedures (using 7.5 mg finely chopped hair), and the analysis method liquid chromatography coupled with tandem mass spectrometry (LC–MS/MS) analysis were conducted by the Dresden lab and have been previously described by Gao et al. (2013). For HC, HCn, HDHEA, and HProg, intra-assay coefficients of variation were 8.7%, 7.2%, 10.2%, and 12.5%, respectively, and inter-assay coefficients of variation were 12.6%, 8.6%, 13.8%, and 18.2%, respectively. According to a most recent study (Gomez-Gomez and Pozo 2020) using liquid chromatography tandem mass spectrometry for the determination of steroid profile in hair, the LC/MS–MS method is considered precise if CVs are below 15% and 20% for medium-to-high and low quality controls (QC), respectively.

### Quality criteria for the assessment of hair sample quality

The Society of Hair Testing (SoHT) (Cooper et al. 2012) published guidelines for drug testing in hair which are in line with the recommendations published by several research groups over recent years (e.g., Greff et al. 2019; Pragst and Balikova 2006; Wennig 2000; Wester and van Rossum 2015). Based on these guidelines, recommendations, and our own experience, two raters evaluated the hair samples regarding nine criteria using a dichotomous scale (‘0’ does not fulfill criterion, ‘1’ = fulfills criterion) (Table 1). Criterion 1 (‘collection site’) was evaluated by the two raters separately, based on digital photographs and sketches created by laypersons and research staff as part of the documentation during the hair collection. In the case of missing information or disagreement between the two raters, a conservative rating was applied (i.e., coded with ‘0’). The remaining criteria (2–9) were simultaneously evaluated by the two raters. Each criterion was multiplied by a weighting factor (1, 2, or 3), reflecting the degree of potential detriment to the quality of the hair sample if not fulfilled. In brief, weighting factor ‘1’ was applied to label the hair sample for identification purposes (criterion 9). Weighting factor ‘2’ indicates factors that facilitate hair processing before analysis and thus enhance the quality of analysis (criteria 2, 5, 6, 7). Lastly, weighting factor ‘3’ reflects factors that may increase the risk of inaccuracy, potential errors, and variability, such as incorrect collection site, visibly shifted cut-off of hair sample, and ambiguous proximal end of the hair sample (criteria 1, 3, 4, 8). Finally, a total score was calculated by summing up the weighted scores of all nine criteria (0–21).

**Table 1:**
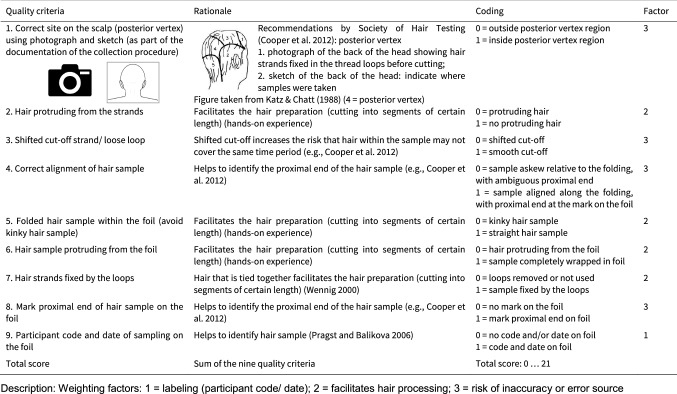
Criteria of hair quality, including rationale, coding, and factor

### Statistical analysis

First, using boxplot analysis to reduce biases, we excluded three, five, and one extreme value(s) (> 3 SD) in HC, HDHEA, and HProg, respectively. One participant was excluded from HProg analyses because one measurement (HOME sample) was below the detection limit. Another participant was excluded from all hair steroid analysis due to an insufficient amount of hair in the HOME sample collected by a layperson (6.2 mg instead of the required 7.5 mg hair for extraction). Since log-transformation did not result in normal distribution of the hair steroid data, non-parametric statistics were computed using raw data. Specifically, Spearman correlation (*rs*) and Wilcoxon signed-rank tests (*Z*) were used to test associations and differences in hair steroid concentrations between samples collected by laypersons (HOME) and research staff (LAB), respectively (hypotheses 1.1, 1.2). For hair sample quality, weighted total scores between HOME and LAB samples were compared using Wilcoxon signed-rank test (*Z*) (hypothesis 1.3). The impact of instruction on the magnitude of absolute difference in hair steroid concentration between samples collected by laypersons and research staff (ΔHair steroid =|HOME—LAB|), hair sample quality (weighted total score), overall evaluation of instructions, and performance confidence was statistically tested using Mann–Whitney *U* tests (*U*) (hypotheses 2.1–2.4). Multiple regression analysis and subsequent partial Spearman correlations (Conover 1999) were conducted to investigate whether laypersons’ characteristics and HOME collection circumstances were associated with ΔHair steroid and hair sample quality (hypotheses 3.1–3.3). All analyses were conducted using IBM SPSS 26.0 (Chicago, IL, USA).

## Results

### Hair steroid concentrations and quality of hair samples collected by laypersons and trained research staff (1.1, 1.2, 1.3)

**Fig. 1:**
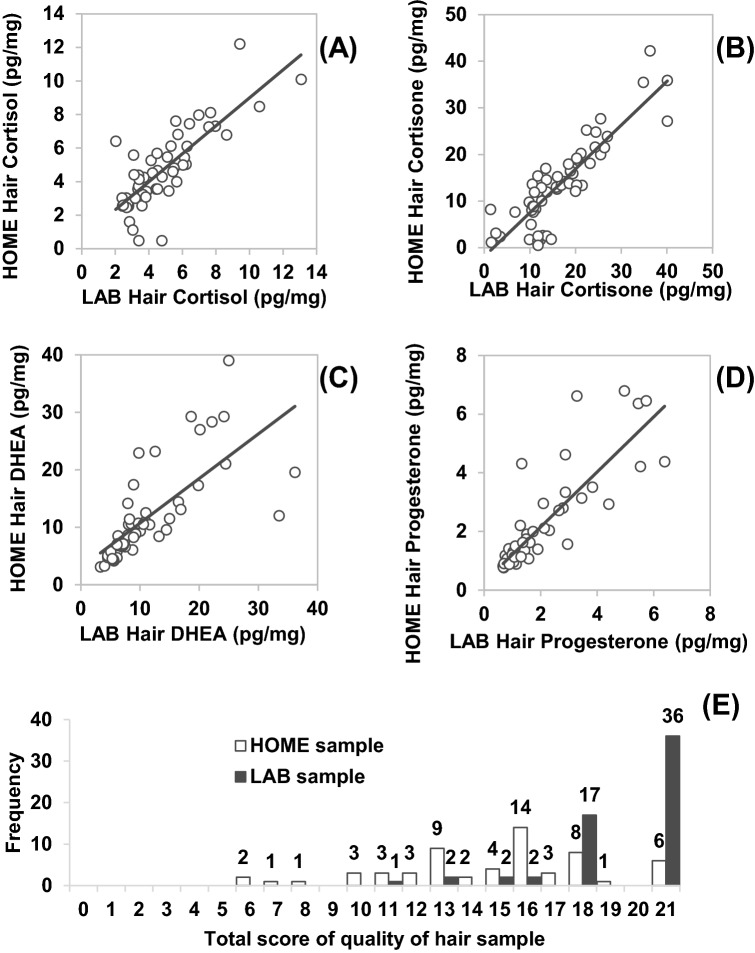
**A** Scatterplot depicting the association between hair cortisol concentration (HC), collected at home by laypersons and at the laboratory by trained research staff; **B** Scatterplot depicting the association between hair cortisone concentration (HCn), collected at home by laypersons and at the laboratory by trained research staff; **C** Scatterplot depicting the association between hair DHEA concentration (HDHEA), collected at home by laypersons and at the laboratory by trained research staff; **D** Scatterplot depicting the association between hair progesterone concentration (HProg), collected at home by laypersons and at the laboratory by trained research staff; **E** Quality of hair samples collected at home and at the laboratory, indicated by weighted total score (0–21)

Correlation analyses revealed significant positive associations between HOME and LAB samples for hair cortisol (*rs* = 0.76, *p* < 0.001, *n* = 56; see Fig. 1A), hair cortisone (*rs* = 0.84, *p* < 0.001,* n* = 59; see Fig. 1), hair DHEA (*rs* = 0.89, *p* < 0.001, *n* = 54; see Fig. 1), and hair progesterone (*rs* = 0.88, *p* < 0.001, *n* = 57; see Fig. 1). The majority of steroid concentrations did not significantly differ between hair samples collected at home and at the laboratory: hair cortisol (HC) (*Z* = − 0.80, *p* = 0.43; HOME: *Md* = 4.27; 0.48–12.21 pg/mg; LAB: *Md* = 4.17, 2.03–13.07 pg/mg), hair DHEA (HDHEA) (*Z* = − 0.63, *p* = 0.53; HOME: *Md* = 9.13; 3.13–39.02 pg/mg; LAB: *Md* = 8.62, 3.29–36.18 pg/mg), and hair progesterone (HProg) (*Z* = − 1.71, *p* = 0.09; HOME: *Md* = 1.39; 0.76–6.79 pg/mg; LAB: *Md* = 1.36, 0.67–6.39 pg/mg). However, a significant difference emerged for hair cortisone (HCn) (*Z* = − 4.37, *p* < 0.001), with overall lower cortisone concentrations in hair samples collected at home (*Md* = 13.03; 0.50–42.26 pg/mg) than in those collected at the laboratory (*Md* = 14.72, 1.42–40.10 pg/mg). We repeated the statistical analyses using the original dataset without previous exclusion of extreme values, which yielded comparable results (see Suppl. S2). There was a significant difference in the quality of hair samples (*Z* = − 6.01, *p* < 0.001), indicated by higher total score values in LAB (*Md* = 21, 11–21) compared to HOME samples (*Md* = 16, 6–21) (see Fig. 1E).

### The impact of instructions on hair steroid concentrations, hair quality, and performance confidence *(Hypotheses 2.1, 2.2, 2.3, 2.4)*

Written instructions alone (A) and written/video-based instructions (B) did not significantly differ in terms of the magnitude of absolute hair steroid differences between samples collected by laypersons and research staff (ΔHC: *U* = 333.00, *p* = 0.31; (A): *Md* =|0.64|; |0.01| to |2.97|), *n* = 28; (B): *Md* =|0.85|; |0.13| to |4.39| *n* = 28; ΔHCn: *U* = 368.00, *p* = 0.31; (A): *Md* =|2.70|; |0.08| to |11.32|, *n* = 29; (B): *Md* =|3.17|; |0.18| to |12.96|¸ *n* = 30; ΔHDHEA: *U* = 359.00, *p* = 0.96; (A): *M*d =|1.35|; |0.00| to |16.61|, *n* = 25; (B): *Md* =|1.01|; |0.07| to |21.45|, *n* = 29; ΔHProg: *U* = 379.00, *p* = 0.68; (A): *Md* =|0.18|; |0.01| to |3.33|, *n* = 27; (B): *Md* =|0.25|; |0.00| to |2.01|, *n* = 30). There was no significant difference between instructions in terms of the quality of the HOME hair samples (*U* = 421.00, *p* = 0.67; (A): *Md* = 16; 6–21, *n* = 30, (B): *Md* = 15.5; 10–21, *n* = 30). Laypersons who received both written and video-based instructions (B) rated the instructions as significantly more helpful and comprehensible than those who solely received written instructions (A) (*U* = 265.50, *p* < 0.01; (A): *Md* = 4; 2–5, *n* = 29, (B): *Md* = 5; 2–5, *n* = 30). However, the format of instructions had no significant effect on how confident laypersons felt about their own performance (*U* = 378.00, *p* = 0.39; (A): *Md* = 6.8; 0.4–9.4, *n* = 29, (B): *Md* = 7.1; 2.6–9.5, *n* = 30).

### The impact of HOME collection circumstances and layperson characteristics on hair steroid concentrations and quality of samples (*Hypotheses 3.1, 3.2, 3.3*)

Neither ΔHC, ΔHCn, nor hair sample quality was associated with characteristics of HOME collection circumstances (familiarity with the participant, confidence in own performance) and layperson’s characteristics (conscientiousness) (see Tables 2, 3, 6). However, the magnitude of absolute difference in hair DHEA concentrations between HOME and LAB samples (ΔHDHEA) was significantly associated with lower self-reported confidence in performance (Table 4). Familiarity with the participant was significantly associated with the magnitude of absolute difference in hair progesterone concentrations between HOME and LAB samples (ΔHProg); however, the regression model was not significant (Table 5). HOME collection circumstances and layperson characteristics did not affect the association between HOME and LAB hair steroid concentrations (Tables 2, 3, 4, 5, 6).

**Table 2:** Magnitude of absolute difference in hair cortisol concentrations between samples collected by laypersons and research staff (ΔHC) and potential confounders

	ΔHC (*n* = 55)			Partial correlation *rs* between HOME and LAB HC
	*B*	*SE*	*t*	
Constant	0.053	0.883	0.060	
Familiarity with the participant	0.046	0.076	0.610	0.76*** (*n* = 56)
Performance confidence	0.056	0.062	0.897	0.77*** (*n* = 55)
NEO-FFI conscientiousness	0.007	0.017	0.380	0.76*** (*n* = 56)

Description: *R*^2^_adjust_ = − 0.03; *F*(3, 54) = 0.48; *p* = 0.71; ΔHC = magnitude of absolute difference in hair cortisol concentrations between samples collected by laypersons and research staff; *B* = unstandardized beta estimate; *SE* = standard error; *t* = *t* statistic; ***p < 0.001

**Table 3:** Magnitude of absolute difference in hair cortisone concentrations between samples collected by laypersons and research staff (ΔHCn) and potential confounders

	ΔHCn (*n* = 58)			Partial correlation *rs* between HOME and LAB HCn
	*B*	*SE*	*t*	
Constant	2.367	3.059	0.774	
Familiarity with the participant	0.112	0.263	0.426	0.84*** (*n* = 59)
Performance confidence	0.283	0.215	1.318	0.86*** (*n* = 58)
NEO-FFI conscientiousness	− 0.036	0.059	− 0.607	0.84*** (*n* = 59)

Description: *R*^2^_adjust_ = − 0.01; *F*(3, 57) = 0.74; *p* = 0.54; ΔHCn = magnitude of absolute difference in hair cortisone concentrations between samples collected by laypersons and research staff; *B* = unstandardized beta estimate; *SE* = standard error; *t* = *t* statistic; *** *p* < 0.001

**Table 4:** Magnitude of absolute difference in hair DHEA concentrations between samples collected by laypersons and research staff (ΔHDHEA) and potential confounders

	ΔHDHEA (*n* = 53)			Partial correlation *rs* between HOME and LAB HDHEA
	*B*	*SE*	*t*	
Constant	9.353	3.766	2.483*	
Familiarity with the participant	− 0.129	0.326	− 0.395	0.90*** (*n* = 54)
Performance confidence	− 0.773	0.265	− 2.912**	0.90*** (*n* = 53)
NEO-FFI conscientiousness	− 0.004	0.074	− 0.057	0.89*** (*n* = 54)

Description: *R*^2^_adjust_ = 0.10; *F*(3, 52) = 2.89; *p* = 0.045; ΔHDHEA = magnitude of absolute difference in hair DHEA concentrations between samples collected by laypersons and research staff; *B* = unstandardized beta estimate; *SE* = standard error; *t* = *t* statistic; *p < 0.05, **p < 0.01, ***p < 0.001

**Table 5:** Magnitude of absolute difference in hair progesterone concentrations between samples collected by laypersons and research staff (ΔHProg) and potential confounders

	ΔHProg (*n* = 56)			Partial correlation *rs* between HOME and LAB HProg
	*B*	*SE*	*t*	
Constant	− 0.195	0.671	− 0.291	
Familiarity with the participant	0.116	0.055	2.118*	0.88*** (*n* = 57)
Performance confidence	0.014	0.046	0.297	0.87*** (*n* = 56)
NEO-FFI conscientiousness	− 0.012	0.012	− 1.036	0.88*** (*n* = 57)

Description: *R*^2^_adjust_ = 0.04; *F*(3, 55) = 1.75; *p* = 0.17; ΔHProg = magnitude of absolute difference in hair progesterone concentrations between samples collected by laypersons and research staff; *B* = unstandardized beta estimate; *SE* = standard error; *t* = *t* statistic; *p < 0.05. ***p < 0.001.

**Table 6:** Quality of HOME hair samples and potential confounders

	Quality of HOME hair samples (*n* = 59)		
	*B*	*SE*	*t*
Constant	11.794	3.134	3.763***
Familiarity with the participant	− 0.234	0.270	− 0.865
Performance confidence	0.300	0.221	1.359
NEO-FFI conscientiousness	0.094	0.061	1.551

Description: *R*^2^_adjust_ = 0.03; *F*(3, 58) = 1.59; *p* = 0.20; *B* = unstandardized beta estimate; *SE* = standard error; *t* = *t* statistic; ***p < 0.001

## Discussion

This study aimed to compare hair samples collected by laypersons (at the participant’s home) and trained research staff (at the laboratory) with regard to hair steroid concentrations (cortisol, cortisone, DHEA, progesterone) and the quality of hair samples. A further study goal was to investigate whether and how the format of instructions, familiarity between participant and layperson, layperson’s confidence in his/her own performance and conscientiousness may affect hair outcome measures. In summary, our findings suggest a significant and high positive association between HOME and LAB hair steroid concentrations, and a higher quality rating of LAB compared to HOME hair samples. The format of instructions did not affect the magnitude of difference between HOME and LAB hair steroid concentrations, quality of HOME hair sample, and laypersons’ confidence in their performance; however, written combined with video-based instructions were perceived as more helpful and comprehensive than written instructions alone. Overall, characteristics of HOME collection and of laypersons (familiarity with the participant, performance confidence, and conscientiousness) did not predict the hair quality of HOME hair samples and the majority of hair steroids (Δhair steroids). Only low self-reported confidence in performance was significantly associated with the magnitude of absolute difference in hair DHEA concentrations between HOME and LAB samples (ΔHDHEA).

The first finding is in line with the results of previous studies using HC (Enge et al. 2020; Ouellet-Morin et al. 2016), although the current study revealed a slightly weaker (but nevertheless high) HOME and LAB HC association than did previous studies (*rs* = 0.76 vs. *r* = 0.91 and *r* = 0.84). Despite the obvious methodological differences between the studies (e.g., type of correlation coefficient, analysis method for cortisol measurement with specific measurement range and error), it is possible that several additional factors might have affected the strength of the correlation. For example, the exclusion of outliers resulted in a relatively narrow range, which may in turn have reduced the inter-individual variation in HCC and the strength of the correlation. Immunoassays, as used by the two previous studies (Enge et al. 2020; Ouellet-Morin et al. 2016), tend to overestimate values, in contrast to the gold-standard LC–MS/MS (Russell 2015); thus, even small differences in low values measured by LC–MS/MS might have resulted in higher within-individual variations compared to differences in high values measured by immunoassays. Related to the differences in measurement sensitivity and accuracy between analysis methods, the two previous studies using immunoassays (Enge et al. 2020: intra-assay and inter-assay CV below 8%; Ouellet et al. 2016: intra-assay CV: 5.39%) reported slightly lower coefficients of variation (CVs) compared to LC–MS/MS in our study (cortisol: 8.7% and 12.6% for intra- and inter-assay CV, respectively). Although the CVs were considered acceptable for LC–MS/MS (Gomez-Gomez and Pozo 2020), the precision of the analysis method might have an impact on the estimation of values (measurement error), and in turn, may affect the correlation due to the within-individual variation. The within-individual variance in HCC might be further increased by possible deviations from the ideal site on the scalp (‘posterior vertex’) (Cooper et al. 2012), as there is some evidence of lower intra-individual variations in HCC within the posterior vertex than within or between other collection regions (Li et al. 2012; Sauve et al. 2007). Although participants were instructed to cut hair samples as close as possible to the scalp, a slight shift of the reflected time window in the hair segment due to an inaccurate cut (e.g., LeBeau et al. 2011; Pragst 2004) cannot be entirely excluded. While electric razors may facilitate scalp-near hair sampling, it is likely that participants prefer scissors over razors, as they may perceive razors as threatening and even dangerous due to the relatively large and sharp blade on common razors (i.e., increased risk of leaving a visible mark a visible mark or cutting the scalp). As a side note, slight unintentional deviations from the collection standards may constitute a general issue and risk in hair studies, but are unfortunately often ignored and not addressed, presuming ‘perfect’ hair collection. In summary, various methodological and statistical factors might have contributed to the slightly weaker but still strong association. Unfortunately, it is not possible to compare the correlations for the other hair steroids (HCn, HEDA, HProg) with previous findings due to the lack of studies in this regard.

With the exception of hair cortisone (HCn), hair steroid concentrations (HC, HDHEA, and HProg) did not significantly differ between samples collected by laypersons and research staff. We have no plausible explanation for this preliminary finding with respect to hair cortisone. Single methodological studies allow only preliminary conclusions and should not be over-interpreted. Thus, replication studies including various hair steroids are of utmost importance.

As expected, the quality of hair samples collected at the laboratory was significantly higher than that of hair samples collected at home, possibly due to the past hands-on experience of the trained research staff. The addition of the video-based instruction had no beneficial effect over the standard written and illustrated instructions, as indicated by hair outcome measures (Δhair steroids and quality of HOME hair samples). The current findings suggest that the format of instructions has no impact on laypersons’ confidence in their performance. Laypersons who received video instructions along with the written instructions evaluated the instructions as slightly, but significantly, more helpful and comprehensible than did laypersons who solely received written instructions. Following the video instructions may require less cognitive resources and time than reading written instructions, which may have contributed to this positive overall rating in favor of the instruction video. The video-based format might be particularly suitable for younger laypersons who use modern electronic media on a daily basis. Taken together, the current findings provide further support for the notion that with the help of detailed instructions, laypersons are able to collect high-quality hair samples for a reliable hair steroid measurement.

The current study also considered HOME collection circumstances and personality of the laypersons collecting the hair sample, and whether these factors may affect hair outcome measures. Overall, laypersons’ self-reported familiarity with the participant and their conscientiousness were neither associated with the difference in HOME and LAB hair steroid concentrations nor with the hair sample quality. This is in line with the study by Enge et al. (2020), who found no impact of personality traits, such as conscientiousness, on HC differences. In the current study, low self-reported performance confidence was associated with the magnitude of the difference between HOME and LAB DHEA concentrations. Given that this association was not evident in the other hair steroids, this finding should be interpreted with caution. Although it is initially plausible that low confidence in performance may reflect poor hair sampling, and may thus affect the measurement of hair steroids, the current data provide no evidence for an association between performance confidence and hair sample quality. One may conclude there are no further beneficial characteristics required of the individual conducting hair collection as long as this individual is able (as well as confident and willing) to follow the instructions.

### Limitations and directions for future research

Study specific sample characteristics need to be taken into account for the interpretation and critical discussion of the study results. The main limitation lies in the unequal sex distribution of the participants in this study, which decreases the gender representativeness and generalizability of the findings. This imbalance towards females is unfortunately common in studies using hair lengths of at least 3 cm as an inclusion criterion, simply because males tend to have shorter hair. Future studies should replicate our findings but might also investigate smaller time windows (e.g., as measured in 1- or 2-cm hair samples), thus increasing the likelihood of including male participants who would otherwise not fulfill the minimum hair length criterion. A strength of this study can be found in the assessment and exploration of the collection process at HOME and the characteristics of the layperson who collected the hair from the participant, which enables researchers to gain a better understanding of whether and how person-related and situational factors may have an impact on hair outcome measures. Future studies are needed to replicate the findings of this and previous studies. Through the use of new media such as interface websites or apps for smartphone devices, laypersons can receive a step-by-step guide for hair collection (written and video-based instructions, help menu, interactive feedback functions) and provide information about hair collection on data-protected online platforms (e.g., documentation, hair characteristics questionnaire).

## General conclusion

Taken together, HOME hair collection by instructed laypersons offers a promising alternative to laboratory and home visits, particularly in epidemiological studies for which researchers aim to reach large and representative populations simultaneously, despite challenging living situations and responsibilities (e.g., limited mobility, flexibility) or environmental circumstances (e.g., quarantine due to pandemics, warzones, disaster zones, hospitalization, imprisonment). To conclude, the current study provides further evidence for a reliable assessment of steroids in hair samples collected by instructed laypersons. Accordingly, HOME hair collection conducted by laypersons might be considered if a direct researcher-participant interaction is not possible, or is only possible with great effort on either side.

## Supplementary Information

Below is the link to the electronic supplementary material.

Supplementary file1 (DOCX 50 KB)

## Footnotes

1 Please note that HOME and LAB samples refer to hair samples collected by laypersons and trained research staff, respectively. Thus, the terms referring to HOME and laypersons can be used interchangeably, as can the terms referring to LAB and research staff.
