# Supplementary material for: HOME vs. LAB hair samples for the determination of long-term steroid concentrations: a comparison between hair samples collected by laypersons and trained research staff
Source: J Neural Transm (Vienna). 2021 Jul 20;128(9):1371–80. doi: 10.1007/s00702-021-02367-3 (PMC8423693; doi:10.1007/s00702-021-02367-3)
Supplement: Supplementary file 1 — Supplementary file1 (DOCX 50 KB) [file 702_2021_2367_MOESM1_ESM.docx]

**Suppl S1: Questionnaires**

Table Suppl. S1.1: Summary of questionnaires answered by participants, laypersons, and trained professionals

| Questionnaire | Description | Participant | Layperson | Trained professional |
| --- | --- | --- | --- | --- |
| Sociodemographic | Sex, age, marital status, children, housing condition (number of residents, type of housing), education, occupation, employment, income | Paper-pencil | Paper-pencil | Sex, age |
| Hair characteristics | Sex, age, height, weight, natural hair color, hair curvature, frequency of hair washes, treatment (e.g., coloring, perm, hair straightening), usage of hair products (e.g., shampoo, conditioner, hair spray) | Paper-pencil | n/a | n/a |
| Screening Scale of Trier Inventory for the Assessment of Chronic Stress (TICS - SSCS) (Schulz et al. 2004) | Chronic stress within the last 3 months | Online Survey | n/a | Online Survey |
| Perceived Stress Scale (PSS) (Cohen et al. 1983; Klein et al. 2016) | Perceived stress within the last 4 weeks | Online Survey | n/a | Online Survey |
| Berlin Social Support Scale (BSSS) (Schulz and Schwarzer 2003) | Social support | Online Survey | n/a | Online Survey |
| Resilience Scale (RS) (Schumacher et al. 2005; Wagnild and Young 1993) | Resilience (personality trait) | Online Survey | n/a | Online Survey |
| Multidimensional Fatigue Inventory (MFI) (Schwarz et al. 2003; Smets et al. 1995) | Fatigue (subscales: general fatigue, physical fatigue, reduced activity, reduced motivation, mental fatigue) | Online Survey | n/a | Online Survey |
| Patient Health Questionnaire MDD (PHQ) (Gräfe et al. 2004; Kroenke et al. 2001) | a) depression severity, b) fulfilling criteria for a major depression, c) fulfilling criteria for another depressive disorder | Online Survey | Paper-pencil | Online Survey |
| NEO Five-Factor Inventory (NEO-FFI) (Borkenau 1991; Costa and McCrae 1992) | Big Five: neuroticism, extraversion, openness to experiences, agreeableness, conscientiousness | Online Survey | Paper-pencil | Online Survey |

Description: Brief PHQ-9 = Brief Patient Health Questionnaire; BSSS = Berlin Social Support Scale; MFI = Multidimensional Fatigue Inventory; NEO-FFI = NEO Five-Factor Inventory; PSS = Perceived Stress Scale; RS = Resilience Scale; TICS-SSCS = Screening Scale of Trier Inventory for the Assessment of Chronic Stress.

Table Suppl. S1.2: Descriptive summary of questionnaires answered by participants and laypersons

| Description | Participant | Layperson |
| --- | --- | --- |
| Sex: Females (%)  Age in years: M ± SD (min - max)  BMI in kg/m^2^: M ± SD (min - max)  Handedness  Right-handed (%)  Left-handed (%)  Two-handed (%)  Missing (%) | 43 (72%)  23.6 ± 3.9 (18-34)  21.8 ± 2.2 (17.63 – 28.36) | 36 (60%)  25.8 ± 8.7 (18-64)  51 (85)  4 (6.7)  4 (6.7)  1 (1.7) |
| Self reported hair color  Blond  Dark blond – light brown (%)  Brown, black (%)  Missing (%)  Hair curvature  Straight (%)  Wavy (%)  Curly (%)  Hair washes per week: M ± SD (min - max)  Treatment (coloring, perm, hair straightening): n (%) | 15 (25)  21 (35)  21 (35)  3 (5)  38 (63.3)  18 (30)  4 (6.7)  4.8 ± 1.7 (2-7)  8 (13.3) |  |
| TICS - SSCS^a^: M ± SD (min - max) | 17.66 ± 6.34 (5 – 31) |  |
| PSS^a^: M ± SD (min - max) | 13.86 ± 4.98 (3 – 24) |  |
| BSSS^a^: M ± SD (min - max) | 3.75 ± 0.31 (3 – 4) |  |
| RS^a^: M ± SD (min - max) | 63.60 ± 7.40 (48 – 72) |  |
| MFI^a^: M ± SD (min - max)  General fatigue  Physical fatigue  Reduced activity  Reduced motivation  Mental fatigue | 9.97 ± 2.82 (4 – 16)  7.79 ± 2.50 (4 – 13)  8.60 ± 3.12 (4 – 15)  7.57 ± 3.23 (4 – 16)  9.45 ± 2.72 (4 – 16) |  |
| Brief PHQ-9:  Depression Severity: M ± SD (min - max)  Major Depression: number of cases  Other depressive disorder: number of cases | 4.57 ± 2.45 (0 – 12)  0  2 | 4.45 ± 2.93 (0 – 14)  1  3 |
| NEO-FFI: M ± SD (min - max)  Neuroticism  Extraversion  Openness to experiences  Agreeableness  Conscientiousness | 17.57 ± 7.18 (2 – 36)  30.07 ± 5.89 (13 – 43)  33.13 ± 5.56 (20 – 42)  34.93 ± 5.78 (17 – 46)  34.28 ± 7.89 (16 – 47) | 18.45 ± 6.96 (6 – 36)  29.05 ± 5.82 (11 – 39)  31.97 ± 7.30 (12 – 45)  33.05 ± 5.93 (17 – 45)  32.40 ± 7.85 (9 – 45) |
| Familiarity with the participant: M ± SD (min - max) | 8.23 ± 1.76 (1.76 – 10) |  |

Description: Brief PHQ-9 = Brief Patient Health Questionnaire; BSSS = Berlin Social Support Scale; MFI = Multidimensional Fatigue Inventory; NEO-FFI = NEO Five-Factor Inventory; PSS = Perceived Stress Scale; RS = Resilience Scale; TICS-SSCS = Screening Scale of Trier Inventory for the Assessment of Chronic Stress; ^a^ n = 58.

**References (questionnaires and scales)**

Borkenau POF (1991) Ein Fragebogen zur Erfassung fuenf robuster Persoenlichkeitsfaktoren. A questionnaire for determining five stable personality factors. Diagnostica 37:29-41

Cohen S, Kamarck T, Mermelstein R (1983) A global measure of perceived stress. J Health Soc Behav 24:385-396

Costa PT, McCrae RR (1992) Revised NEO Personality Inventory (NEO-PI-R) and NEO Five-Factor Inventory (NEO-FFI) professional manual. Psychological Assessment Resources, Odessa

Gräfe K, Zipfel S, Herzog W, Löwe B (2004) Screening psychischer Störungen mit dem "Gesundheitsfragebogen für Patienten (PHQ-D). Diagnostica 50:171-181

Klein EM et al. (2016) The German version of the Perceived Stress Scale - psychometric characteristics in a representative German community sample. BMC Psychiatr 16:159. <https://doi.org/10.1186/s12888-016-0875-9>

Kroenke K, Spitzer RL, Williams JB (2001) The PHQ-9: validity of a brief depression severity measure. J Gen Intern Med 16:606-613. <https://doi.org/10.1046/j.1525-1497.2001.016009606.x>

Schulz P, Schlotz W, Becker P (2004) TICS Trierer Inventar zum chronischen Stress. Hogrefe, Göttingen

Schulz U, Schwarzer R (2003) Soziale Unterstützung bei der Krankheitsbewältigung. Die Berliner Social Support Skalen (BSSS). Diagnostica 49:73-82

Schumacher JLKGT, Strauss B, Braehler E (2005) Die Resilienzskala - Ein Fragebogen zur Erfassung der psychischen Widerstandsfaehigkeit als Personmerkmal. The Resilience Scale - A questionnaire for the assessment of resilience as a personality characteristic. ZKPP 53:16-39

Schwarz R, Krauss O, Hinz A (2003) Fatigue in the general population. Onkol 26:140-144. <https://doi.org/10.1159/000069834>

Smets EM, Garssen B, Bonke B, De Haes JC (1995) The Multidimensional Fatigue Inventory (MFI) psychometric qualities of an instrument to assess fatigue. J Psychosom Res 39:315-325. <https://doi.org/002239999400125O>

Wagnild GM, Young HM (1993) Development and psychometric evaluation of the Resilience Scale. J Nurs Meas 1:165-178

Suppl. S 1.3: Relationship between participant (hair donor) and layperson (hair collector)

The following information will be provided by the **person who collect the hair sample** (hair collector).

[Nachfolgende Informationen sind bitte durch die **Person, die die Haarprobe entnimmt (HaarsammlerIn)**, zu beantworten.]

**Please indicate what your relationship is to the person whose hair was collected by you (hair donor)?** (multiple answers possible).

[**In welchem Beziehungsverhältnis stehen Sie zur Person, der Sie die Haarprobe entnommen haben (HaarspenderIn)?** (Mehrfachantworten möglich)]

|  | No  [nein] | Yes  [ja] |  |
| --- | --- | --- | --- |
| We are related to each other.  [Wir sind miteinander verwandt.] | 🞎 | 🞎 | If yes, please specify (for example, if you are the mother, please write “mother”)  [wenn ja, bitte spezifizieren Sie (z.B. wenn Sie die Mutter sind, schreiben Sie bitte „Mutter“)] |
| We are in an intimate relationship (e.g., liaised, married).  [Wir sind in einer partnerschaftlichen Beziehung (z.B. liiert, verheiratet).] | 🞎 | 🞎 |  |
| We live together (one household).  [Wir wohnen im gleichen Haushalt.] | 🞎 | 🞎 |  |
| We are friends.  [Wir sind miteinander befreundet.] | 🞎 | 🞎 |  |
| We are professionally acquainted.  [Wir sind beruflich miteinander bekannt.] | 🞎 | 🞎 | If yes, please specify (for example, colleagues, fellow students)  [wenn ja, bitte spezifizieren Sie Ihr berufliches Verhältnis (z.B. Kollegen, Kommilitonen):] |
| Other  [Anderes] | 🞎 | 🞎 | If yes, please specify:  [wenn ja, bitte spezifizieren Sie:] |

**How familiar are you with the person whose hair you collected (hair donor)?**

Please mark a cross through the line in the position that best describes your **response**.

[**Wie vertraut sind Sie mit der Person, der Sie eine Haarprobe entnommen haben (HaarspenderIn)?**

Bitte zeichnen Sie an der Stelle auf der Linie ein Kreuz ein, die Ihrer **persönlichen Einschätzung** am meisten entspricht.]

Not at all Very

[Gar nicht] [sehr]

Suppl. S1.4: Evaluation of the instruction material by layperson

Please evaluate the **instruction material**.

[Bitte beurteilen Sie das **Instruktionsmaterial**.]

|  | Very untrue  [trifft  überhaupt  nicht zu] |  |  |  | Very true  [trifft  voll und  ganz zu] |
| --- | --- | --- | --- | --- | --- |
| Overall, the instruction material was helpful and comprehensible.  [Das Informationsmaterial war insgesamt hilfreich/ verständlich.] | 1 | 2 | 3 | 4 | 5 |
| The written instructions ▶„Instruction for hair sampling“ (p. 2-3) were helpful and comprehensible.  [Die schriftliche Instruktion ▶„Anleitung zur Haarprobenentnahme“ (S. 2-3) war hilfreich/ verständlich.] | 1 | 2 | 3 | 4 | 5 |
| ▶„Tips for hair sampling“ (p. 4) were helpful and comprehensible.  [Die ▶„Hinweise für das Sammeln von Haarproben“ (S. 4) waren hilfreich/ verständlich.] | 1 | 2 | 3 | 4 | 5 |
| The video clip (CD) was helpful and comprehensible.  [Das ▶Video (CD) war hilfreich/ verständlich.] | 1 | 2 | 3 | 4 | 5 |

Overall, how **confident** do you feel that you have collected the hair sample properly according to the instructions? Please mark a cross through the line in the position that best describes your **response**.

[Wie **sicher** fühlen Sie sich insgesamt, dass Sie die Haarprobe korrekt entnommen haben, so wie es in der Instruktion beschrieben war? Bitte zeichnen Sie an der Stelle auf der Linie ein Kreuz ein, die Ihrer **persönlichen Einschätzung** am meisten entspricht.]

Not confident Very confident

[unsicher] [sehr sicher]

🡪 If you felt less confident, please briefly elaborate in your own words. What can we improve?

[🡪 Falls Sie sich etwas unsicher fühlen, bitte beschreiben Sie dies kurz in Ihren eigenen Worten. Was können wir noch verbessern?]
